# Efficient Catalytic Conversion of Polysulfides by Biomimetic Design of “Branch-Leaf” Electrode for High-Energy Sodium–Sulfur Batteries

**DOI:** 10.1007/s40820-020-00563-6

**Published:** 2021-01-05

**Authors:** Wenyan Du, Kangqi Shen, Yuruo Qi, Wei Gao, Mengli Tao, Guangyuan Du, Shu-juan Bao, Mingyang Chen, Yuming Chen, Maowen Xu

**Affiliations:** 1 Key Laboratory of Luminescence Analysis and Molecular Sensing (Southwest University), Ministry of Education, School of Materials and Energy, Southwest University, Chongqing, 400715 People’s Republic of China; 2 Beijing Computational Science Research Center, Beijing, 100193 People’s Republic of China; 3 Center for Green Innovation, School of Materials Science and Engineering, University of Science and Technology Beijing, Beijing, 100083 People’s Republic of China; 4 College of Environmental Science and Engineering, Fujian Normal University, Fuzhou, 350007 People’s Republic of China

**Keywords:** Co nanoparticles, Sodium–sulfur batteries, Branch-leaf, biomimetic

## Abstract

Rechargeable room temperature sodium–sulfur (RT Na–S) batteries are seriously limited by low sulfur utilization and sluggish electrochemical reaction activity of polysulfide intermediates. Herein, a 3D “branch-leaf” biomimetic design proposed for high performance Na–S batteries, where the leaves constructed from Co nanoparticles on carbon nanofibers (CNF) are fully to expose the active sites of Co. The CNF network acts as conductive “branches” to ensure adequate electron and electrolyte supply for the Co leaves. As an effective electrocatalytic battery system, the 3D “branch-leaf” conductive network with abundant active sites and voids can effectively trap polysulfides and provide plentiful electron/ions pathways for electrochemical reaction. DFT calculation reveals that the Co nanoparticles can induce the formation of a unique Co–S–Na molecular layer on the Co surface, which can enable a fast reduction reaction of the polysulfides. Therefore, the prepared “branch-leaf” CNF-L@Co/S electrode exhibits a high initial specific capacity of 1201 mAh g^−1^ at 0.1 C and superior rate performance.

**Electronic Supplementary Material:**

The online version of this article (10.1007/s40820-020-00563-6) contains supplementary material, which is available to authorized users.

## Highlights

- 3D “branch-leaf” biomimetic design is proposed for high-performance Na-S batteries.
- The conductive “branch” can ensure adequate electron and electrolyte supply with the “leaf” can catalyze the conversion of polysulfides.
- DFT calculation reveals that the Co nanoparticles can enable fast reduction reaction of the polysulfides;
- The prepared CNF-L@Co/S electrode exhibits a high initial specific capacity of 1201 mAh g^−1^ at 0.1 C and superior rate performance.

**Electronic Supplementary Material:**

The online version of this article (10.1007/s40820-020-00563-6) contains supplementary material, which is available to authorized users.

## Graphical abstract

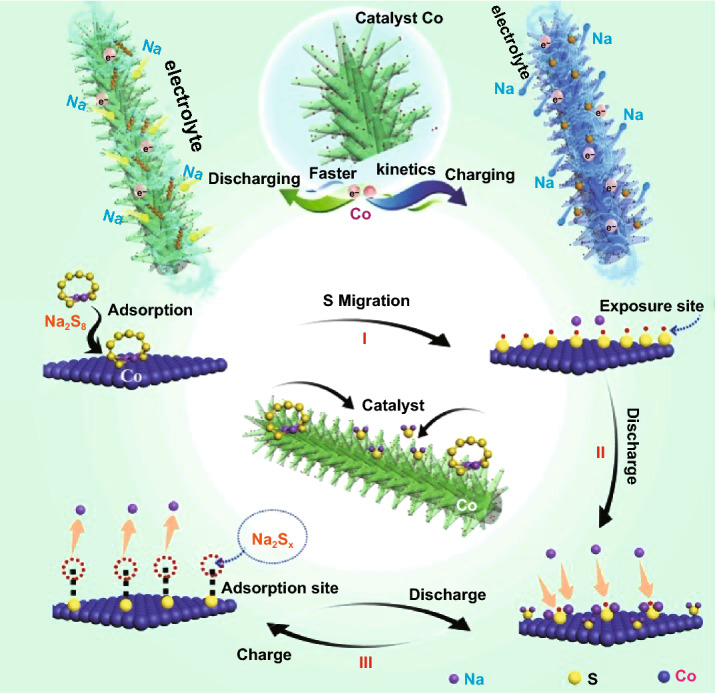

## Introduction

To store renewable energy sources, which possess the characteristics of intermittency and randomness, rechargeable battery techniques have been widely investigated by the electrical energy storage community [1, 2]. Among the rechargeable battery techniques, room temperature sodium–sulfur (RT Na–S) batteries have emerged as one of the most promising “green technique” due to their high theoretical specific capacity (1675 mAh g^−1^) and eco-friendly of sulfur [3, 4]. However, the intrinsic electronic/ionic insulation of sulfur and its reduction products is extremely unsatisfactory to achieve the full utilization of the active material (sulfur) [5, 6]. Moreover, the shuttling behaviors of dissolved intermediate sodium polysulfides (NaPSs) would result in poor cycling stability [7, 8].

To alleviate these drawbacks, great efforts have been devoted by researchers to design and synthesize favorable S-based cathodes with unique compositions and structures for building high performance RT Na–S batteries [9, 10]. The most common strategy is to physically confine sulfur in various carbon materials, such as micro/mesoporous carbon, 2D graphene, hollow carbon nanotubes, and conductive polymers [11, 13]. However, such non-/weak-polar carbon materials only offer weak interactions toward polar NaPSs, which are insufficient to suppress the shuttling effect over a long lifespan, finally making against the long-term cycle stability of sulfur cathode. Therefore, other polar functional groups, such as polar metal-based compounds like oxides, nitrides, carbides, and hydroxides, have been developed as cathode hosts of sulfur to adsorb polar NaPSs by forming strong polar interaction [14, 15]. Although these materials can strengthen the interaction between NaPSs and sulfur cathodes, most of them still suffer from low sulfur utilization, primarily due to the slow redox kinetics of polysulfides conversion, large radius of Na^+^, and its inferior reactivity with sulfur. The sluggish kinetics of polysulfides conversion also increase the accumulation of soluble intermediate NaPSs in the cathode area and their inevitable diffusion into the electrolyte from the cathode to anode forms insoluble solid Na_2_S_2_/Na_2_S [16, 17]. Recently, the catalysis of the polysulfide conversion has been reported as an effective method to fundamentally address the loss of sulfur by accelerating the redox kinetics of polysulfides conversion [18]. Since “polysulfide catalysis” is a relatively new concept, the exploration of more catalyst candidates with high activity and low cost, as well as a more efficient catalytic polysulfide conversion is a priority area of research. Currently, various transition metals are widely used to speed up the electrochemical reaction rates, according to results from Li–S battery [19–23]. For instance, wang’s group reported that monodisperse cobalt atoms embedded in grapheme can trigger the surface-mediated reaction of Li polysulfides since the Co–N–C coordination center serves as a bifunctional electrocatalyst. This conception leads to the rapid formation of short-chain polysulfide and ultimately Li_2_S and can also enhance the conductivity of the cathode [24]. A similar concept has been demonstrated in RT Na–S batteries. Yang’s group reported that Cu nanoparticles loaded in mesoporous carbon can be utilized to immobilize sulfur and polysulfides, while a novel Cu foam current collector is able to activate sulfur electroactivity. In our group, we designed and synthesized a 3D host consisting of carbon fiber concatenated nickel hollow spheres for accelerating the electrochemical kinetics of RT Na–S batteries. Each nickel atom exhibits a significant catalytic effect in the conversion of polysulfide to accelerate the reaction kinetics of liquid intermediates, finally alleviating the shuttle effect to some extent. Similarly, Zhang et al. reported a highly effective sulfur host with atomic Co (including SA Co and Co clusters) supported in the micropores of hollow carbon (HC) nanospheres as a cathode for RT Na–S batteries [15]. In their study, they have fundamentally studied the mechanism for the improvement of electrochemical performance and found that the maximized atomic utilization could optimize the multiple functions of cobalt metal enhancing sulfur conductivity, activating sulfur reactivity, and immobilizing sulfur and sodium polysulfides [15]. The results of the latter work also show that the metal Co nanoparticles possess metallic characteristics and even super catalytic activity, which largely beneficial to facilitate the redox reaction kinetics and increase the sulfur utilization efficiency for Na–S batteries [24, 25]. Despite such promising results, the charge–discharge mechanism of cobalt as a catalyst in RT Na–S batteries is not clear and needs further investigation. In addition, traditional sulfur cathode, which is prepared based on slurry-casting technique, with large amounts of inactive materials (including conductive agent, polymer binder, and metal current collectors) would significantly decrease the energy density of batteries [27]. In contrast, self-supported binder-free cathode hosts, such as carbon nanofibers can avoid this disadvantage, thus have attracted much interest from researchers. Electrospinning is one strategy to effectively produce freestanding nanofibers with excellent mechanical integrity and outstanding conductivity [28, 29]. Therefore, it is rational, yet challenging, to introduce catalyst into a free-standing sulfur electrode to maximize the various functions of a polarized sulfur host and achieve extraordinary performance for RT Na–S batteries. Metal–organic framework(MOFs) are often used as a sacrificial template to convert as-prepared hierarchical structures into nanoporous carbon while retaining the desirable properties. In addition, nitrogen-containing MOFs will yield N-doped porous carbons upon thermal carbonization, which could greatly enhance the catalytic activity of porous carbons for electrochemical storage applications. The zeolitic imidazolate framework (ZIF) is type of MOF with a multiple structure containing mesoporous and microporous pore sizes. ZIF is particularly of interest due to its simple fabrication process and nitrogen-containing precursor [30, 31].

**Fig. 1:**
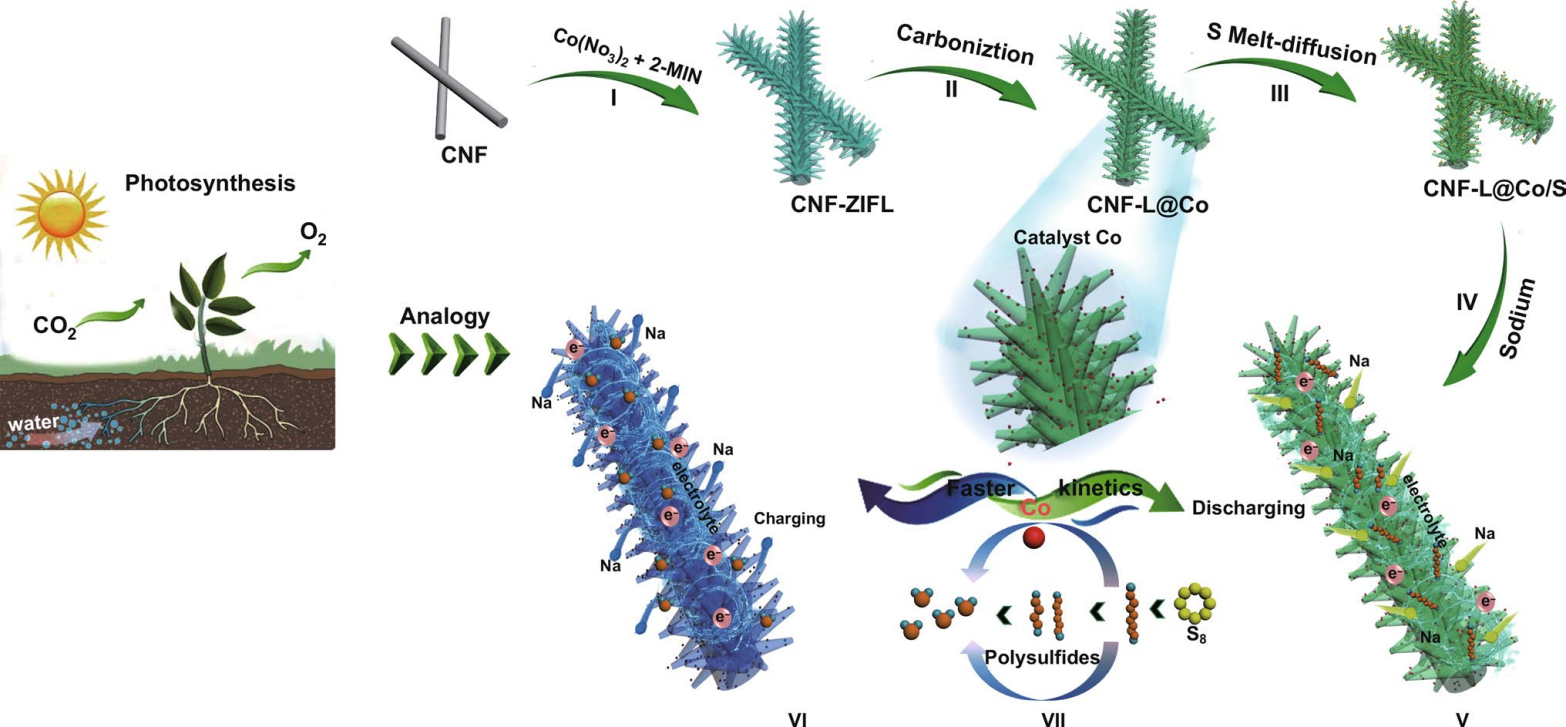
Schematic of the preparation of CNF-L@Co/S and the catalytic mechanism of CNF-L@Co/S in RT Na–S batteries

The cooperation of branches and leaves to convert solar energy into biological energy through photosynthesis is one of the most effective catalytic systems found in nature. In this system, branches continuously supply water and nutrients while leaves absorb solar energy simultaneously; both are critical for efficient photosynthesis (Fig. 1). Remarkably, the electrochemical catalytic process is similar to photosynthesis observed in trees. In electrochemical catalysis, active sites accelerate the reaction rate, while current collectors are responsible for the rapid transmission of electrons. Motivated biologically, a “branch-leaf” cathode host was designed in this work.

Herein, in order to achieve high performance RT Na–S batteries with increased energy density, a self-support “branch-leaf” structural cathode (CNF-L@Co/S) was designed and synthesized by integrating well-conductive carbon fibers as the “branches” via electrospinning technique and carbon coated Co nanoparticles as the “leaves”. The conductive “branch”can ensure adequate electron and electrolyte supply and physically confine S/NaPSs. The carbon coated Co “leaves” can anchor polysulfides and then catalyze their conversion. Finally, attributed to the synergistic effect of the “branch-leaf” structure, the as-prepared CNF-L@Co/S cathode exhibits a high reversible capacity of 1201 mAh g^−1^ (602.5 Wh Kg^−1^) at 0.1 C, excellent cycling stability, and superior rate capability. In addition, DFT calculations demonstrate that a unique Co–S–Na molecular layer formed on the surface of Co nanoparticles, which can enable rapid redox reaction of the polysulfides.

## Experimental Section

### Materials and Methods

#### Preparation of CNF Matrix

PAN nanofibers were fabricated through an electrospinning technique. A precursor solution was first prepared by dissolving 1000 mg PAN (MW = 150,000) into 10 mL N,N-dimethylformamide (DMF) with vigorous stirring, forming a pale yellow viscous homogenous solution. Then, the solution was sucked into a 10 mL plastic syringe with a needle and positioned 18 cm away from the collector. When a high voltage of 30 kV was applied to the system, PAN nanofibers were generated and collected with a feeding rate of 0.0008 mm s^**−**1^. The as-spun white nanofiber films were stabilized at 60 °C for 12 h in a vacuum oven.

#### Preparation of Leaf-like Zeolitic Imidazolate Framework (ZIF-L) on CNF

The CNF/ZIF-L precursor was synthesized using a solution method. First, 582 mg Co(NO_3_)_2_·6H_2_O and 1300 mg 2-methylimidazole (2-MIM) were dissolved in 40 mL deionized water. Then, cobalt nitrate solution was quickly added to the 2-MIM solution and mixed under agitation for 1 min at room temperature to obtain a purple solution. Next, a piece of PAN nanofiber film was directly immersed in the mixture solution and aged for 50 min under room temperature. After rinsing the sample with deionized water and allowing it to air dry, a purple ZIF on fiber film was obtained.

#### Preparation of CNF-L@Co/S Composite

The obtained purple ZIF on fiber film was placed in a porcelain boat and first annealed at 350 °C for 20 min with a heating rate of 2 °C min^**−**1^. Then, the temperature increased to 800 °C at a rate of 5 °C min^**−**1^ maintained for 2 h under the protection of Ar atmosphere to obtain the CNF-L@Co compound. Finally, the sample pressed into small round disks with size of 1 × 1 cm^2^ using a table press. For uniform sulfur impregnation, the prepared CNF-L@Co compound was completely soaked in the S/CS_2_ solution (10 mg mL^−1^) using a pipette then dried in an airing chamber. Finally, the composite was heat-treated at 155 °C around the melting point of sulfur for 12 h to obtain the uniform composite. For comparison, to remove the Co species, the acquired black electrodes were then immersed in 6 M HCl aqueous solution via a hydrothermal reaction at 130 °C for 2 h. After being washed with deionized water to remove the acid, CNF-L@Co/S was successfully converted into CNF-L/S. CNF/S film was also produced following same method but without ZIF.

### Electrochemical Measurements

The free-standing CNF-L@Co/S and CNF/S films were cut into 10 mm disks and directly used as electrodes without any other additives. The electrochemical test conditions were the same as those in our previous paper [39]. 1 M of sodium perchlorate (NaClO_4_, Sigma Aldrich, > 98%) in a mixture of ethylene carbonate/diethyl carbonate (EC/DEC, 1:1 v/v%) was used as the electrolyte in a dosage of 90 mL. The total weight of the cathode was − 2.5 mg. The specific capacities were estimated based on the loading of sulfur in the cathodes (1–1.2 mg cm^−2^).

## Results and Discussion

### Synthesis and Characterization

Motivated biologically, a “branch-leaf” cathode host for Na–S batteries was designed in this work. The “branches” consisting of uniform carbon nanofibers (CNF) were prepared by simple electrospinning of PAN (polyacrylonitrile Fig. 1). Then, a uniform shell of ZIF with leaf-like morphology was formed on the PAN nanofibers through a solution deposition method at room temperature (Fig. 1 I). To preserve morphology and achieve high electronic conductivity simultaneously, the as-prepared CNF-ZIF composite was treated through a two-step carbonization process, during which the “leaves” were formed by the accumulation of carbon coated Co nanoparticles (the final product is marked as CNF-L@Co in Fig. 1 II). Finally, sulfur was successfully impregnated into the CNF-L@Co composite (denoted as CNF-L@Co/S) by a melt-diffusion process (Fig. 1 III).

In the as-proposed unique “branch-leaf” structure, the catalytic “leaves” (Co) on the highly conductive “branches” (CNF) have been constructed as Na–S batteries cathode. As shown in Fig. 1 VII, the internal interwoven CNF “branch” with plentiful pores and satisfactory electronic conductivity can supply transmission channels for the electrolyte/electron, while the external “leaves” with abundant Co active sites can effectively anchor and convert sodium polysulfides. Therefore, the bio-inspired “branch-leaf” structure CNF-L@Co/S appears highly promising in improving the electrochemical properties of RT Na–S batteries.

**Fig. 2:**
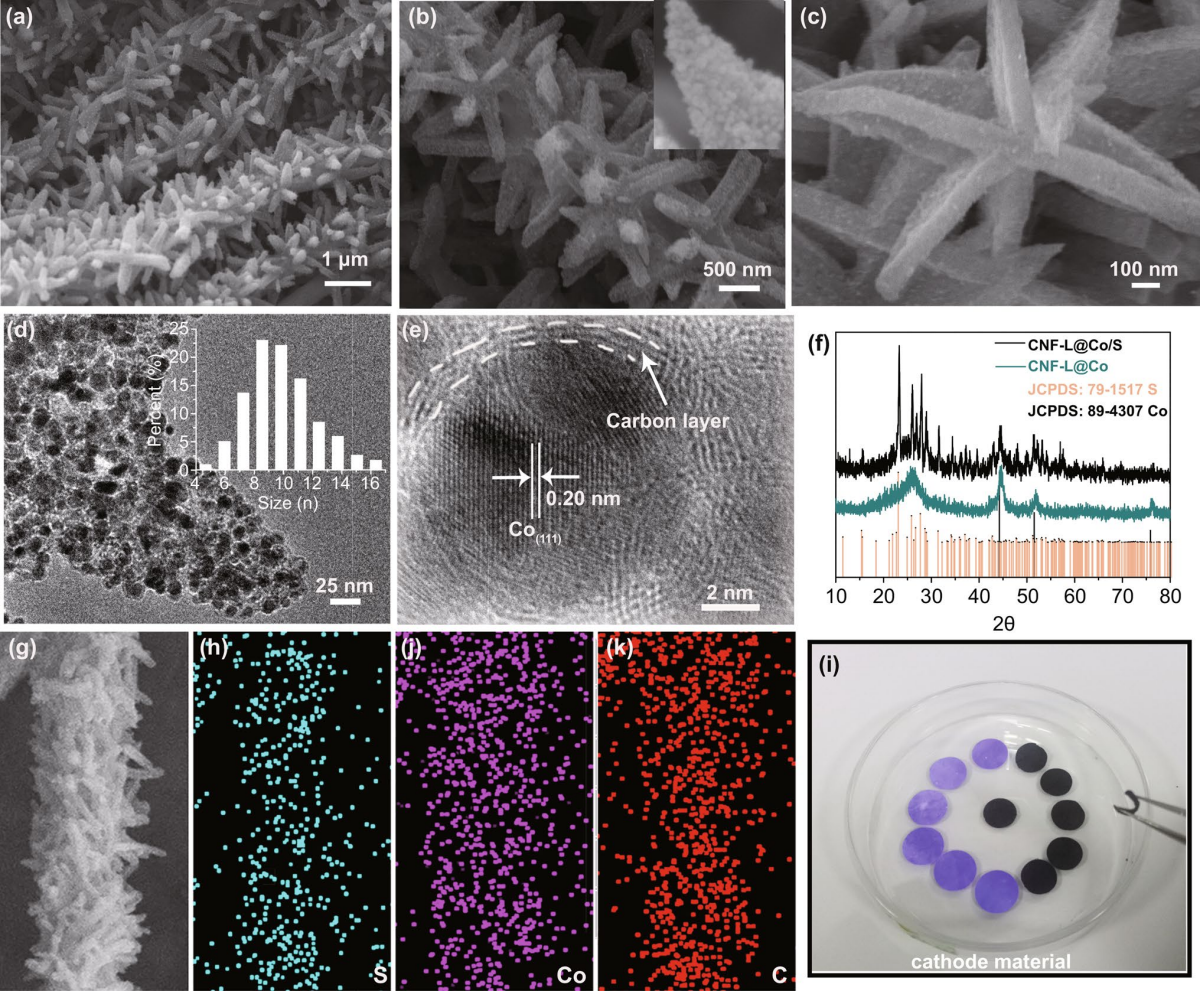
Characterization composites. **a–c** FESEM images of CNF-L@Co, **d, e** TEM images of CNF-L@Co; **f** XRD pattern of CNF-L@Co and CNF-L@Co/S; **g–k** EDS elemental mappings of CNF-L@Co/S, and **i** flexible films of CNF-L@Co/S

As shown in Fig. S1, the diameter of CNFs was determined to be 400 nm. The closely interwoven CNFs are beneficial for the transport of electrons and the infiltration of electrolyte, hence promoting electrochemical reactions. Figure 2a, b show that the carbon nanofibers are uniformly covered by Co nanoparticles forming leaf-like architectures. The FESEM image in Fig. 2c indicates that the length and thickness of the “leaf” are about 800 and 100 nm, respectively. The TEM image in Fig. 2d demonstrates that the Co nanoparticles are uniformly distributed in the ZIF-derived carbon and the diameter of them is less than 18 nm. The obvious fringe with a lattice spacing about 0.20 nm in Fig. 2e corresponds to the (111) lattice plane of Co, indicating the successful transformation of ZIF to Co nanoparticles. In addition, Fig. 2e also shows that the thickness of the carbon layer on Co nanoparticles is around 2 nm. The TGA curve reveals that the content of Co in the CNF-L@Co is about 14.69% (Fig. S3). The X-ray mapping images in Fig. S4 imply that all elements (C, N, and Co) are evenly scatted within the as-prepared composite. X-ray photoelectron spectroscopy (XPS) was employed to study the chemical composition and the oxidation state of the as-prepared CNF-L@Co. As shown in Fig. S5, the N 1 s spectrum displays three strong peaks at binding energies of 401.38, 399.09, and 397.18 eV, corresponding to graphitic-N, pyrrolic-N, and pyridinic-N, respectively [32]. The pyridinic-N and pyrrolic-N have been reported to increase the pseudocapacitance, while graphitic-N increases the overall conductivity. Moreover, the peaks in the C1s spectrum at 283.88, 285.08, and 286.68 can be assigned to C = C *sp*^2^, C–N/C–C* sp*^3^, and C = O species, respectively, which further confirms the N doping in the carbon matrix (Fig. S5a, b) [33]. In the corresponding Raman spectrum in Fig. S6, CNF-L@Co exhibits two broad peaks at 1337 cm^−1^ (D band) and 1575 cm^−1^ (G band), revealing its graphite-like microcrystalline structure, and peaks at 672 cm^−1^ are assigned to the ring structure of Co. In addition, the nitrogen heteroatom substitution into the carbon matrix generates defective sites, thus increasing in the *I*_D_/*I*_G_ intensity [34, 35].

**Fig. 3:**
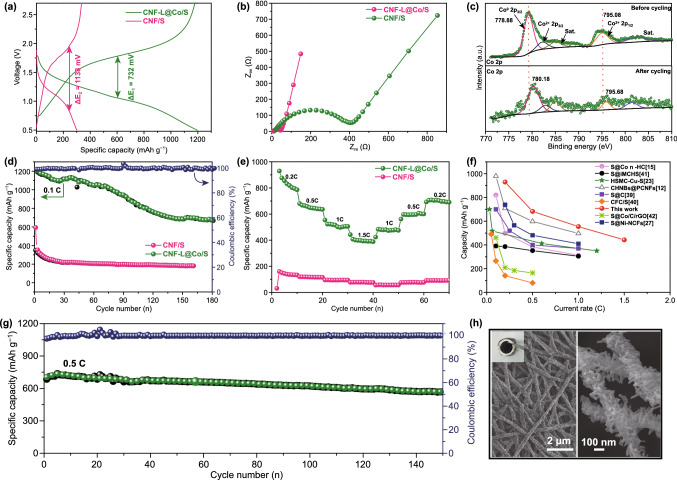
**a** Comparison of discharge/charge curves. **b** Comparison of electrochemical impedance spectra. **c** High-resolution XPS spectra of Co 2p before and after cycling. **d** Cycling performance and Coulombic efficiency of CNF-L@Co/S and CNF/S at 0.1 C. **e** Rate performance of CNF-L@Co/S and CNF/S. **f** Comparison of the rate capabilities with previously reported RT Na-S batteries. **g** Long-term cycling stability of CNF-L@Co/S at 0.5 C, and **h** FESEM images of the CNF-L@Co/S composite after cycling

In Fig. 3d, the pristine Co 2P XPS spectrum can be deconvoluted into Co^0^ (778.88 eV) and Co^2+^ (781.60 eV) which could be derived from the surface oxidation of metallic Co [36, 37]. The Brunauer–Emmett–Teller (BET) surface area and pore volume of CNF-L@Co are 55.662 m^2^ g^−1^ and 0.089 cm^3^ g^−1^, respectively, which is beneficial for the maintenance of sulfur.

Finally, sulfur was successfully impregnated into the CNF-L@Co composite (denoted as CNF-L@Co/S) by a melt-diffusion process. The obtained freestanding CNF-L@Co/S cathode possesses high flexibility and can be directly used as binder-free cathode for RT Na–S batteries. As shown in Fig. 2f, prominent diffraction peaks of orthorhombic sulfur are detected in the CNF-L@Co/S composite accompanied by three strong peaks of Co (JCPDS No. 89–4307), confirming the successful incorporation of sulfur. Structural characterization of the flexible CNF-L@Co/S electrode was also investigated by FESEM and TEM. As shown in Fig. S7, the CNF-L@Co/S composite maintains the original morphology after sulfur loading, and no excess sulfur block can be observed on the surface of the material. The TEM images in Fig. S7d demonstrate that most of the interior space of CNF-L@Co/S become darker, further indicating the sulfur was successfully diffused into the substrate, while a small amount of void space is still left, which could buffer the volume expansion during the discharge/charge process. In addition, the BET surface area and pore volume are substantially reduced to 11.323 m^2^ g^−1^ and 0.028 cm^3^ g^−1^ (Fig. S7f), respectively, revealing the successful accommodation of sulfur into the pores of the CNF-L@Co material. The TGA analysis in Fig. S7c directly confirms that the sulfur content of CNF-L@Co/S is about 45%. The elemental mapping images in Fig. 2g–k verify the homogenous distribution of S, Co, and C, indicating the uniform chemical composition of the CNF-L@Co/S composite material. The stable connection between the “branches” (CNF) and “leaves” (carbon coated Co nanoparticles) supports the efficient electron migration between the conductive carbon substrate and active surface feasible, enabling rapid interfacial reactions. It is worth noting that the mechanical flexibility (shown in Fig. 2i) of the as-obtained CNF-L@Co/S supports its potential as self-supported electrodes for Na–S batteries.

### Electrocatalytic Activity Investigation

The thickness of the free-standing film electrode material is 40.63 μm (Fig. S12a). To demonstrate the effect of the advanced “branch-leaf” structure, electrochemical performances of CNF-L@Co/S and bare CNF/S composite were evaluated by RT Na–S batteries. Figure 3a shows galvanostatic discharge/charge profiles of the CNF-L@Co/S and CNF/S electrodes at 0.1 C. In Fig. 3a, it can be observed that CNF-L@Co/S exhibits a more prominent discharge plateau and reduced polarization (compared to 732 vs 1138 mV of CNF/S). The existence of metallic Co is indeed helpful to dynamically accelerate the electrochemical reactions of sodium polysulfides. Figure. S8a illustrates the chemical interaction on the Co interface. Figure. S8b presents the cyclic voltammogram (CV) curves of both samples at a scan rate of 0.1 mV s^−1^. During the first scan, a pair of redox peaks at 1.8/1.04 V corresponds to the conversion of long-chain polysulfides (Na_2_S_*x*_, *x* = 4–8) to short-chain sodium polysulfides (Na_2_S_2_/Na_2_S) and reversely the oxidation of Na_2_S [13, 38].

Compared with CNF/S, the CNF-L@Co/S electrode manifests a smaller potential hysteresis in its voltage profile as well as smaller gaps between the paired redox peaks in the CV curves in Fig. S9, which serves as pronounced evidence for the catalytic effect of Co nanoparticles. Moreover, such kinetic improvement of the CNF-L@Co/S electrode was further supported by electrochemical impedance spectroscopy (EIS) analysis. As shown in Fig. 3b, the charge-transfer resistance of CNF-L@Co/S electrode is much a smaller than that of the CNF/S electrode (30 vs 198 Ω), indicating its faster charger transfer behavior [39]. Both the doped N and added Co nanoparticles can help improve the electronic conductivity of CNF and the chemical affinity of the as-prepared electrode with NaPSs. As displayed in Fig. 3c, the two characteristic peaks of Co (at 778.88 and 795.08 eV in pristine electrode, attributed to the metallic Co and oxidized Co^2+^) both shift to higher binding energies (− 780.18 and − 795.68 eV) after cycling, indicating the strong chemical interaction between Co and NaPSs [40].

Comparison of cycling performance of the two electrodes is provided in Fig. 3d. The CNF-L@Co/S electrode shows a higher initial capacity than CNF/S at 0.1 C (1201 vs 349 mAh g^−1^), confirming a more efficient polysulfide redox reaction. The unique “leaf-branch” structure ensures effective utilization of the Co “leaf” during electrocatalytic reactions and fast electron transfer by the porous CNF “branch”, which plays a key role in improving the overall electrochemical performance. Moreover, the improved redox kinetics in CNF-L@Co/S is further revealed by its superior rate performance shown in Fig. 3e. The electrode with CNF-L@Co/S delivers a high discharge capacity of about 929.8 mAh g^−1^ at 0.2 C, indicating greater sulfur utilization. Even at increased current rate of 1.5 C, the capacity of the CNF-L@Co/S electrode still remains as high as 442.7 mAh g^−1^, which five times of the CNF/S electrode (77.7 mAh g^−1^). Notably, when the current rate was reduced to 0.2 C, the discharge capacity of the CNF-L@Co/S electrode recovered to 704.6 mAh g^−1^, demonstrating its outstanding stability under high current rates. As shown in Fig. S10a, after etching Co by HCl aqueous solution, the overall morphology of the material remained unchanged, leaving abundant macropores on the surface of CNF-L (Fig. S10b). Furthermore, the supplementary electrochemical properties of the CNF-L/S electrode in Fig. S10c, d, The CNF-L/S electrodes exhibit inferior cycle capabilities relatively of 486.5 mAh g^−1^ at 0.1 C in Fig. S10c. Moreover, it is also suffer slower rate capacity compared to CNF-L@Co/S (Fig. 5d), In addition, the average discharge capabilities of the CNF-L/S at 0.1, 0.2, 0.5, 1, and 1.5 C were 678.5, 657.7, 593.3, 507.8, and 412.1 mAh g^−1^, respectively. The results suggest that the overall electrochemical performance of the CNF-L@Co/S electrode is superior than that of both CNF/S and CNF-L/S electrodes, which confirms the synergistic effect of Co “leave” and CNF “branch” in improving the redox kinetics of polysulfides conversion.

A comparison of the rate capability with that in the state-of-the-art literatures is presented in Fig. 3f and Table S1, which indicates that the rate capability of the prepared CNF-L@Co/S electrode outperforms the majority of reported electrodes [12, 15, 23, 25, 41–44]. Figure 3g shows the long-term cycling performance of the electrode at 0.5 C. The CNF-L@Co/S electrode exhibits a high discharge capacity of 736.8 mAh g^−1^ at the fifth cycle and maintains at 538 mAh g^−1^ after 150 cycles with a low decay rate of 0.17% per cycle. Even at a rather high current rate of 2 C (Fig. S11), CNF-L@Co/S still delivered a high initial discharge capacity of 637.3 mAh g^−1^, which stabilized at 225.7 mAh g^−1^ after 1000 cycles. In addition, the shape and structure of the CNF-L@Co/S can be well retained after cycling (Fig. 3h). These results cooperatively manifest the high sulfur utilization and fast sulfur redox reaction via strong electrocatalytic Co in the “branch-leaf” CNF-L@Co/S electrode. To further validate the advantages of the CNF-L@Co/S cathode, we compared its structure and electrochemical activity with published studies about Co catalytic effect in RT Na–S batteries as shown in Table S2 [15, 27, 45]. It is worth mentioning that the CNFs contribute to the little capacity under the same conditions so it was negligible, as displayed in Fig. S12.

The above results demonstrate that the existence of metallic Co is indeed helpful to accelerate dynamically accelerate the electrochemical reactions of sodium polysulfides, which is illstrated in Fig. S8a. In situ Raman spectroscopy is usually employed to investigate the mechanism of polysulfide formation during discharge and charge. Figure S13 shows the in situ Raman spectra of the CNF-L@Co/S electrode during discharge from 2.4 to 0.5 V followed by recharging to 2.4 V, the curves of particular interest are marked in the corresponding voltage-capacity curve as shown in Fig. S13b. For the pristine electrode, the peaks at 187.3, 337.2, and 495.04 cm^−1^ correspond to the characteristic peaks of S_8_, according to previous literature [16, 46]. After discharged to 2.4 V, the peak of S_8_ become gradually weaker, while a new peak attributable to Na_2_S_4_ appeared at about 426.2 cm^−1^. As the discharge continued, the peak of Na_2_S (at about 463.3 cm^−1^) was observed at 1.0 V, while the intensity kept increasing until it was fully discharged to 0.5 V. In the charge process, the characteristic peaks of Na_2_S_6_, Na_2_S_2_, and Na_2_S_*x*_ can be distinguished at around 342, 477, and 698 cm^−1^, respectively. After charged to 2.8 V, the peaks of both S and deposited Na_2_S_2_ can be detected, indicating that the reaction is partially reversible [34, 47]. Therefore, the in situ Raman patterns reveal that sulfur mainly undergoes a conversion from solid S_8_ into soluble long-chain polysulfide (Na_2_S_*n*_, *n* = 4–8) and a subsequent transformation into short-chain polysulfides into (Na_2_S_2_/Na_2_S), which is consistent with the CV curve (Fig. S8b). Based on the above results, it can be concluded that Co could quickly catalyze the reduction of NaPSs into Na_2_S and thus effectively reduce the dissolution of NaPSs during cycling, contributing to excellent electrochemical performance of the electrode [48].

### DFT Calculations

**Fig. 4:**
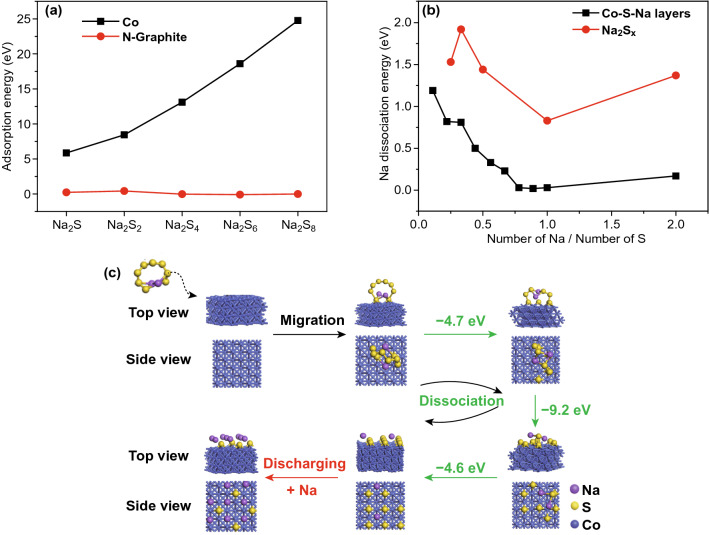
**a** Calculated adsorption energy (eV) for the most favorable adsorption configuration of Na_2_S_*x*_ at Co (111) surface and N-doped graphite at the DFT level. **b** Dissociations for the layered Co–S–Na structures and free Na_2_S_*x*_, *x* = 1–8. **c** Conversion between the molecularly adsorbed Na_2_S_8_ to the dissociatively adsorbed Na2S8 at the Co (111) surface via S migration and embedded sodium

To gain further insight into how the encapsulated Co nanoparticles contribute to the fast kinetics of the CNF-L@Co electrode, DFT calculations were performed. The adsorption energy of Na_2_S_*x*_ (*x* = 1, 2, 4, 6, and 8) polysulfides on an N-doped graphite slab and clean Co (111) slab was calculated (Fig. S14). Since the adsorption of Na_2_S_*x*_ on the N-doped graphite is fairly weak, and thus N-doped graphite will not affect the electronic structure of the Na_2_S_*x*_ adsorbate; the N-doped graphite adsorbed Na_2_S_*x*_ can be considered as free molecules approximately. Comparatively, much stronger Na_2_S_*x*_ adsorption was observed for Co (111) (Fig. 4a), which can be either molecular or dissociative. Among the different types of polysulfides, Na_2_S and Na_2_S_2_ favor the molecular adsorption on clean Co (111), whereas Na_2_S_4_, Na_2_S_6_, and Na_2_S_8_ favor the dissociative adsorption. The molecularly adsorbed Na_2_S_*x*_ remains intact and forms strong Co–S bonds with the surface. In the dissociative adsorption of Na_2_S_*x*_, the S atoms of the molecular Na_2_S_*x*_ migrate onto the Co surface and become dispersed surface S with Co–S bonding (Fig. S15).

In order to better comprehend the process of the dissociative adsorption process, we took Na_2_S_8_ as a typical example. In the dissociative adsorption of Na_2_S_8_, the S atoms in the molecular Na_2_S_8_ migrate onto the Co surface and become gradually dispersed surface single S. The DFT calculation shows that such Na_2_S_8_ decomposition over Co involves a series of exothermal steps (∆H =  − 4.7, − 9.2, and − 4.61 eV, Fig. 4c) and thus is thermally favorable. It is noted that, in the most favorable adsorption configurations for all Na_2_S_*x*_, all S atoms form equal bonds with 4 surface Co atoms (the square Co_4_ surface structure), and none of the Na atoms form bonds with the Co surface (Fig. S15). The strong Co–S bonding accounts for the high adsorption strength of the Na_2_S_*x*_ at the Co surface. Consequently, when increasing the coverage of the surface-adsorbed Na_2_S_*x*_, a surface double layer on top of the Co surface can be formed for all Na_2_S_*x*_ (denoted as Co–S–Na, Fig. S15).

At full coverage, the molecularly adsorbed Na_2_S_2_ appears as a monolayer (ML) of S and a ML of Na (each Na forming equal bonds with the 4 S atoms of the square S_4_ surface structure). The dissociative adsorbed Na_2_S_4_, Na_2_S_6_, and Na_2_S_8_ can be viewed as a ML of S and a fractional ML of Na on top of S-ML; the molecularly adsorbed Na_2_S exhibits the appearance of ½ ML of S and 1 ML of Na.

In order to comprehend the role of the Co–S–Na double-layer adsorption structures, Na dissociation energies (the dissociated Na atom is considered in the solid state) for the layered Co–S–Na structures were first calculated then compared with the Na dissociation energies for the free Na_2_S_x_ molecules (Fig. 4b and the related structures can be found in Fig. S16). The Co–S–Na structures have lower Na dissociation energy than free Na_2_S_*x*_ molecules with the same ratio, as the strong Co–S bonds between the Co surface and S-ML can significantly weaken the S–Na interactions. The energy difference between Co–S–Na and Na_2_S_*x*_ with the same Na:S is −  eV per Na for most of the Na:S range. As a consequence, the Na in Co–S–Na is more susceptible to reduction reactions than that in the Na_2_S_*x*_ molecule, which leads to faster kinetics during the charge process. The Na dissociation energy is also related its reduction potential and charge capacity per Na, i.e., the amount of energy (per Na) can be chemically stored in Co–S–Na or Na_2_S_*x*_ via the charge process. Co–S–Na has a lower charge capacity (by − 1 eV per Na) than Na_2_S_*x*_ with the same Na:S ratio. This implies, energetically, that Co–S–Na lies between Na solid and Na_2_S_*x*_, so Co–S–Na might be an intermediate state for the interconversion between solid Na and Na_2_S_*x*_. In other words, the surface of the Co nanoparticles acts as a catalyst during the charge stage.

**Fig. 5:**
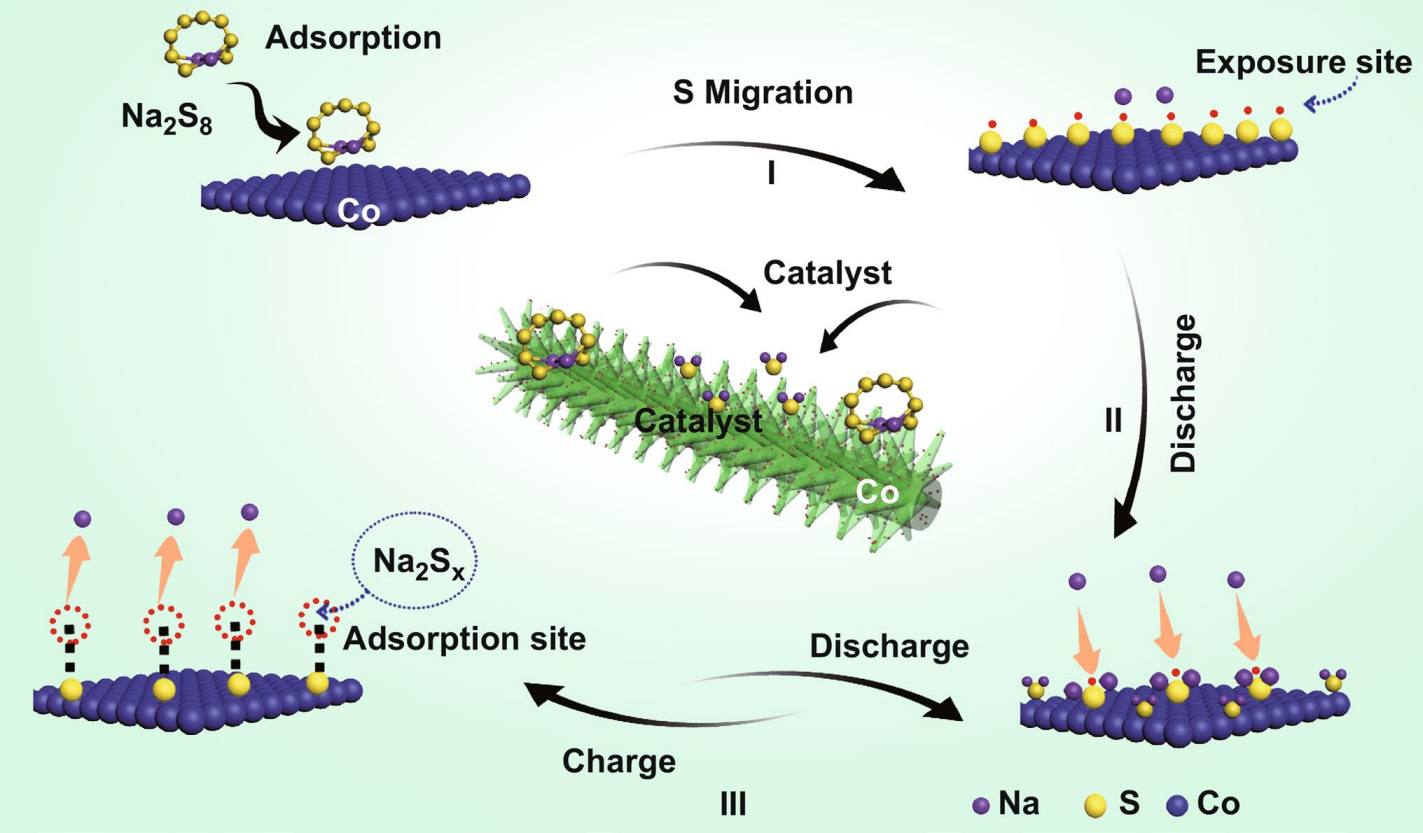
Mechanism for enhancing the Na–S battery performance

Based on the above analyses, we propose the catalytic mechanism for Co nanoparticles induce fast reduction kinetics and enhances the battery performance (Fig. 5). The Co–S–Na structure first forms on the exposed surfaces of Co nanoparticles and acts as a catalyst site during discharge and possibly during charge. This process is predicted to be highly irreversible and thus may only occur during the initial battery runs (Fig. 5c I). During discharging process (Fig. 5c II), the oxidized Na^+^ in the electrolyte enriches near Co, which allows Na^+^ to be directly absorbed onto Co–S–Na to form Na_2_S and other polysulfides in the form of the surface double layer. The locally enriched interface-absorbed Na can further react with Na_2_S_*x*_, resulting in the formation of the final product Na_2_S. The unique Co–S–Na molecular layer structure formed over the Co nanoparticles enables the rapid conversion of polysulfides to form the final Na_2_S product during discharging process.

During charge, Na in Co–S–Na is more susceptible to dissociation than prior to Na in Na_2_S_*x*_ molecule induced by the oxidation of sulfur anion and leads to fast kinetics (Fig. 5c III). The Na-deficient Co–S–Na will form with Na atoms being reduced and removed from the S-ML. The exposed S of the Na-deficient Co–S–Na can capture Na from Na_2_S_*x*_ to fill the vacancies left behind by the removed Na atoms, and a shorter chain polysulfide will be form the remainder of the Na_2_S_*x*_. The Na capture reaction is mildly endothermic, in contrast to the highly endothermic Na reduction of Na_2_S_*x*_, and therefore is viable under the charge condition. The captured Na can be further reduced and removed from Co–S–Na, which ends the old catalytic cycle and starts the new cycle. The Co nanoparticle hence exhibits catalytic performance by breaking the highly endothermic one-step Na reduction reaction of the polysulfides molecules into two mildly endothermic steps, as well as by facilitating the Na capture and oxidation with the S-ML. The consumption rate of electrons is likely to be much faster on Co nanoparticles than the consumption rate on the graphite surface. Such catalytic effects of Co nanoparticles under a large charging current are highly significant to their use in electrodes.

## Conclusion

In summary, a CNF-L@Co/S material with “leaf-branch” structure proposed as a cathode for high performance Na–S batteries. The unique “leaf-branch” structure ensures effective utilization due to Co “leaf” during electrocatalytic reactions and fast electron transfer due to the porous CNF “branch,” which plays a key role in improving the overall electrochemical performance. As a sulfur host, the CNF-L@Co/S cathode exhibits a high reversible capacity of 1201 mAh g^−1^ at 0.1 C, stable long-term cycling stability and superior rate performance. The catalytic effect of Co nanoparticles on sodium polysulfides was verified by various spectroscopic measurements and DFT calculation. Results demonstrated that the Co–S–Na interface layer formed on the surface of Co can alter the pathway for the reduction reaction and promote fast kinetics. Overall, this work provides new insights for the rational design of high performance composite electrodes for RT Na–S batteries.

## Electronic Supplementary Material

Below is the link to the electronic supplementary material.

Supplementary file 1 (PDF 2009kb)
