# Supplementary material for: Efficient Catalytic Conversion of Polysulfides by Biomimetic Design of “Branch-Leaf” Electrode for High-Energy Sodium–Sulfur Batteries
Source: Nanomicro Lett. 2021 Jan 5;13:50. doi: 10.1007/s40820-020-00563-6 (PMC8187676; doi:10.1007/s40820-020-00563-6)
Supplement: Supplementary file 1 — (PDF 2009kb) [file 40820_2020_563_MOESM1_ESM.pdf]

Supporting Information for

Efficient Catalytic Conversion of Polysulfides by Biomimetic Design of "Branch-Leaf" Electrode for High-Energy Sodium-Sulfur Batteries

Wenyan Du[1], Kangqi Shen[2], Yuruo Qi [1], Wei Gao[1], Mengli Tao [1], Guangyuan Du [1], Shu-juan Bao[1], Mingyang Chen [3,] *, Yuming Chen[4,] *, Maowen Xu[1,] *

[1]Key Laboratory of Luminescence Analysis and Molecular Sensing (Southwest University), Ministry of Education, School of Materials and Energy, Southwest University, Chongqing 400715, P. R. China

[2]Beijing Computational Science Research Center, Beijing 100193, P. R. China

[3]Center for Green Innovation, School of Materials Science and Engineering,

University of Science and Technology Beijing, Beijing 100083, P. R. China

Beijing Computational Science Research Center, Beijing 100193, P. R. China

[4]Department of Nuclear Science and Engineering,Department of Materials Science and Engineering,Massachusetts Institute of Technology, Cambridge, Massachusetts 02139, United State

*Corresponding authors. E-mail: xumaowen@swu.edu.cn (Maowen Xu); yumingc@mit.edu (Yuming Chen); mychen@ustb.edu.cn (Mingyang Chen)

S1 Microstructure Characterization

The structures of the samples were characterized by powder X-ray diffraction (XRD, ShimdzuXRD-7000) with Cu Kα radiation. Raman spectra for materials were recorded using an Invia Refl (Renishaw, UK). The morphologies, compositions and microstructures of the composites were analyzed via field-emission scanning electron microscopy (FESEM, JSM-7800F), energy dispersive spectrometry (EDS), and transmission electron microscopy (TEM, JEM-2100), respectively. X-ray photoelectron spectroscopy (XPS) measurements were carried out on a Thermo Scientific ESCALAB 250Xi electron spectrometer. Thermogravimetric analysis was performed using a Thermo Gravimetric Analyzer (TGA, Q50). The analyses for the specific surface area and the size distribution of the products were performed using the Brunauer-Emmett-Teller method (BET, Quantachrome Instruments, USA).

S2 Computational Methods

The adsorption behaviors of Na_2S_x (x=1, 2, 4, 6, 8) polysulfides on CNF-L@Co/S composite were modelled using on first-principles density functional theory (DFT) [S1] carried out by using Vienna ab initio simulation package (VASP) [S2]. The Co nanoparticle region and CNF region of CNF-L@Co were modelled separately, using a Co (111) slab and a N-doped graphite (111) slab respectively. The Co (111) slab is constructed with a 3×3×2 supercell of the (111)-cleaved surface and it contains 4 Co layers and a total number of 72 Co atoms. The N-doped graphite slabs are constructed using a supercell that contains 4 layers and a total number of 72 atoms. One of the 18 surface C atoms of the surface layer is replaced by the N dopant. For both of the slab models, a vacuum of 25 Å is extended to the surface slab to separate the slab and its images. The adsorption behaviors of Na_2S_x at the Co and CNF regions of CNF-L@Co were modelled by interacting the Na_2S_x molecules with the two slabs. Na_2S_x polysulfides, x = 1, 2, 4, 6, and 8 were initially placed at different possible adsorptions sites with several orientations, and optimized until fully relaxed. The adsorption energies E_{ads} for Na_2S_x were evaluated for the optimized adsorption configurations using:

$$E_{ads}(Na_2S_x) = E(Na_2S_x) + E(slab) - E(Na_2S_x@slab) \quad (S1)$$

where $E(Na_2S_x)$, E(slab), and $E(Na_2S_x@slab)$ are the calculated total energies for the Na_2S_x molecule, the surface slab, and the adsorption configuration, respectively. A more positive E_{ads} indicates a stronger adsorption.

The Perdew-Burke-–Ernzerhof (PBE) functional [S3] and the projected augmented wave (PAW) [S4] potentials were used for the DFT calculations. The plane wave cut-off energy was set to be 400 eV. The K-points of the 2D Brillouin zone were sampled using a 3×3×1 Monkhorst-Pack grid [S5] during the structure optimizations and energy computations. The force convergence criterion for the geometry optimization calculations was set to 10^{-4} eV $Å^{-1}$.

The Na dissociation energy for a Na-containing species (denoted as $Na_xS_yCo_z$) related to Co@NPCNFs such as polysulfides and Na/S-adsorbed Co surfaces can be evaluated using:

$$E_{disso}(Na_xS_yCo_z) = E(Na, solid) + E(Na_{x-1}S_yCo_z) - E(Na_xS_yCo_z) \quad (S2)$$

where E is the calculated total energy for each species involved in the Na dissociation reaction, at the DFT level. For the leaving Na atom, we use the per-atom energy of Na solid, E (Na, solid), so that we can compared the Na binding strengths for the different Na-containing species at CNF-L@Co to the solid Na. If x = 2 and z = 0, Eq. 2 yield the dissociation energy for the gas phase polysulfides (Na_2S_x); if $z \neq 0$, the Na dissociation energy is for the Na species associated to the S-covered Co nanoparticles.

S3 Supplementary Tables and Figures

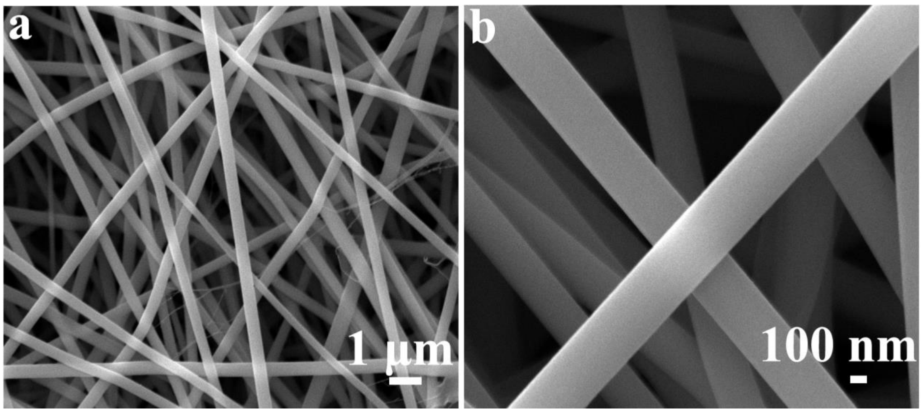

Fig. S1 (**a, b**) FESEM images of the CNF

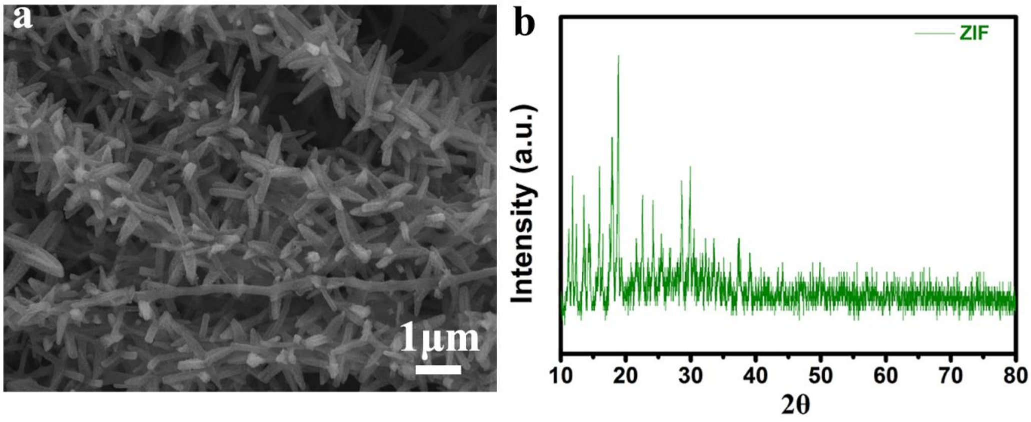

Fig. S2 FESEM images and XRD of the ZIF composite nanofiber before carbonization

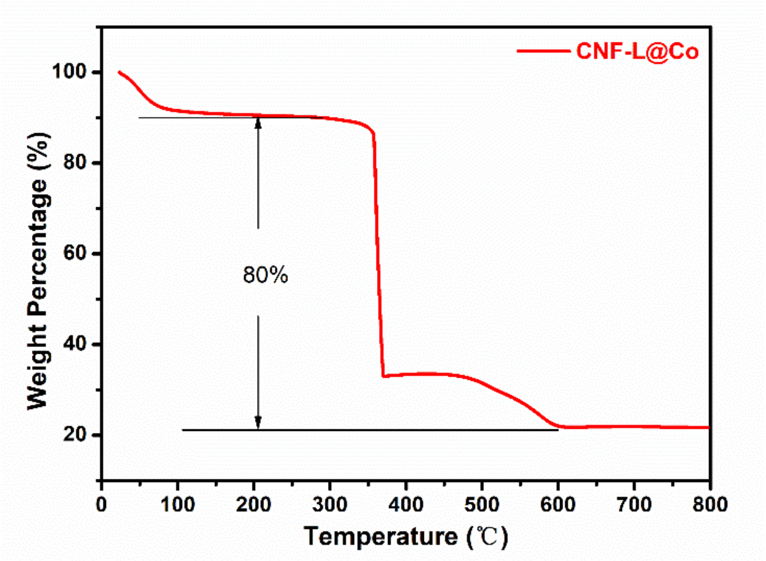

Fig. S3 TGA of the CNF-L@Co in air

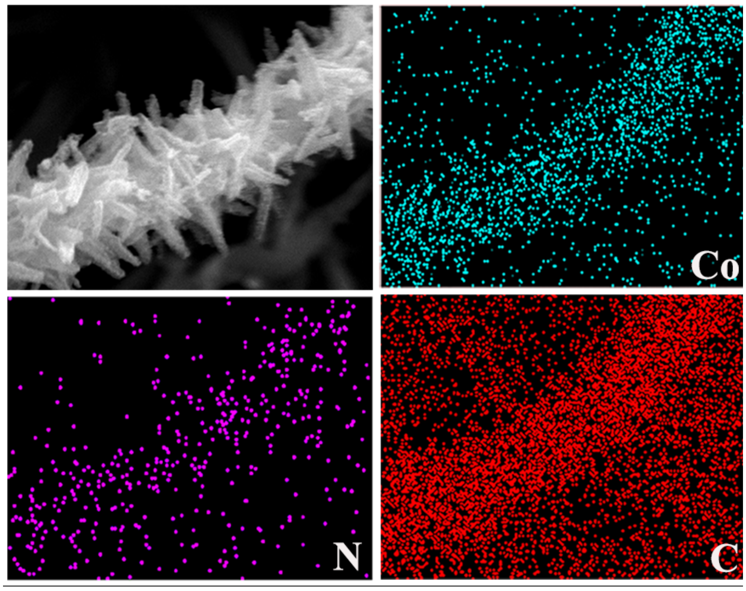

Fig. S4 EDS elemental mappingsof CNF-L@Co composite

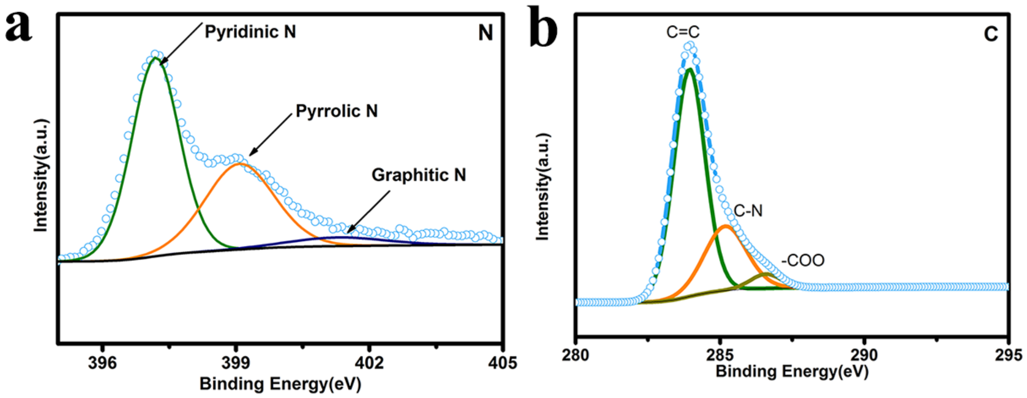

Fig. S5 (**a**) N1s XPS spectrum, (b) C1s XPS spectrum of CNF-L@Co/S

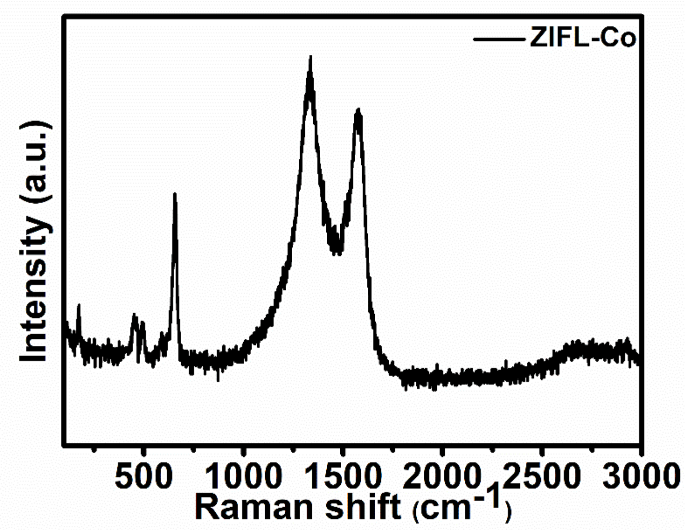

Fig. S6 Raman spectrum of CNF-L@Co

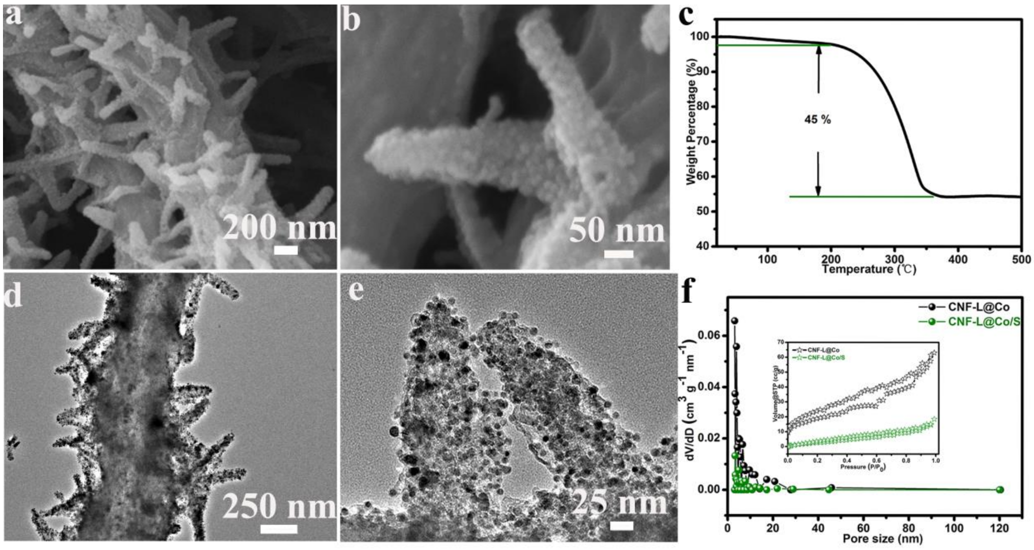

Fig. S7 Characterization of CNF-L@Co/S composite. (**a, b**) FESEM images, (**c**) TGA , (**d, e**) TEM images, (**f**) BET of composite material

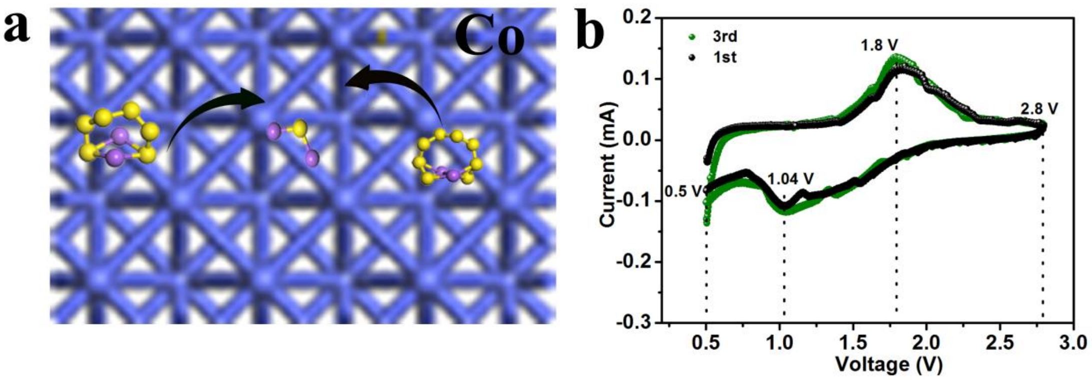

Fig. S8 (**a**) A schematic showing the faster redox kinetics of NaPSs on the Co surface, (**b**) CV curves of CNF-L@Co/S composite

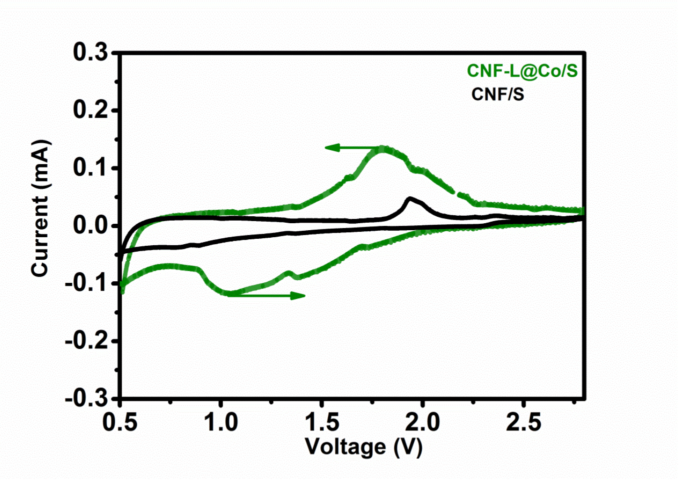

Fig. S9 CV curves of CNF-L@Co/S and CNF/S cathodes at a scan rate of 0.1 mV s^{-1}

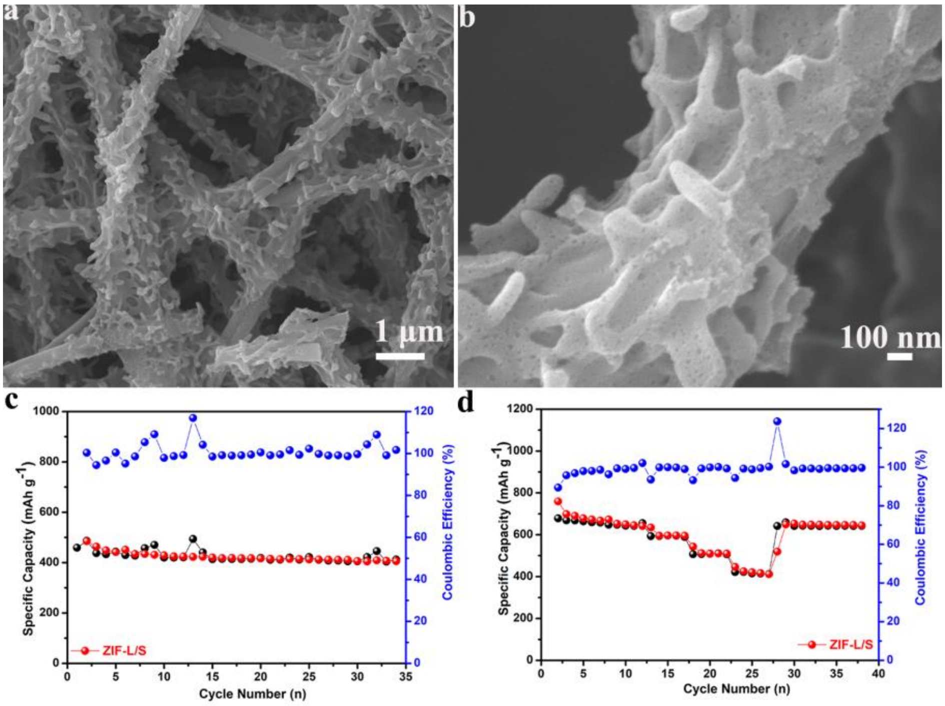

Fig. S10 Characterization composite. (**a, b**) FESEM images of CNF-L. (**c**) Cycling capabilities with the current densityat 0.1 C, and (**d**) Rate performances for CNF-L/S electrode

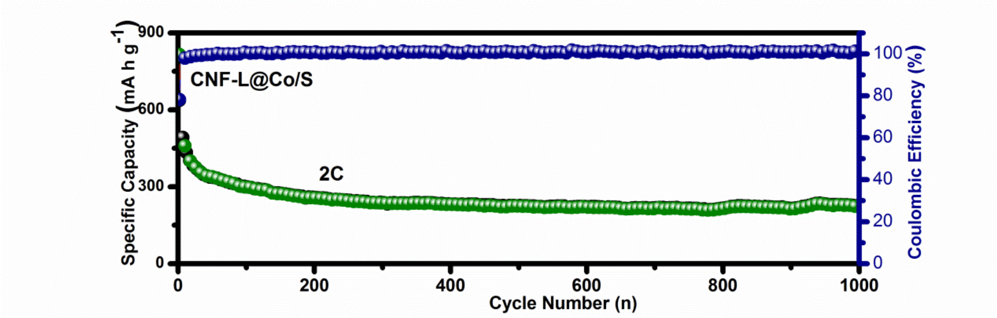

Fig. S11 Long cycling performance of CNF-L@Co/S cathode at 2.0 C

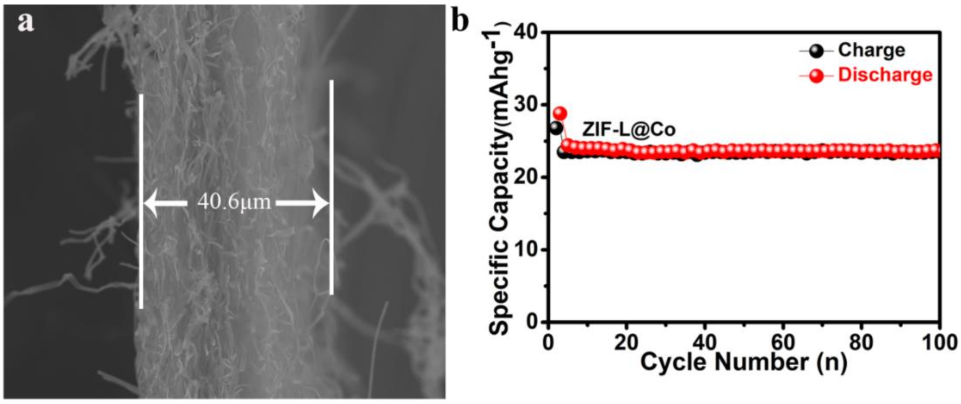

Fig. S12 (**a**) A cross section of the CNF-L@Co/S electrode, (**b**) Cycling performance of CNF-L@Co at 0.1 C

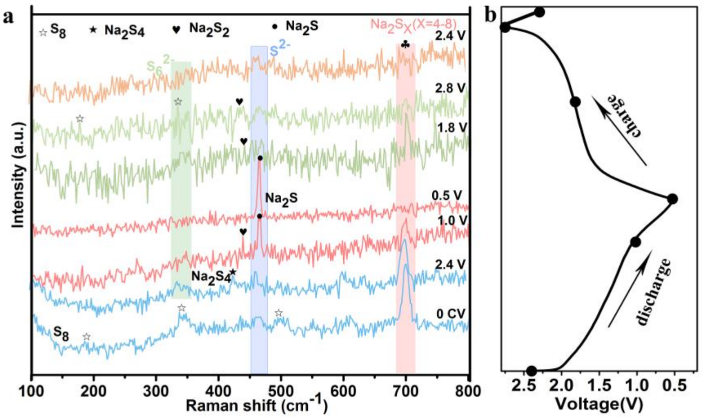

Fig. S13 In situ Raman spectra of the CNF-L@Co/S electrode

Table S1 Cathode composition, sulfur loading, electrolyte composition, and electrochemical performance of RT Na-S batteries with various cathodes that are reported in the literature

**Cathode**	**Sulfur loading**	**Electrolyte**	**Current density/Capacity (mAh g^{-1})**	**Current density/Capacity (mAh g^{-1})**	**Current density/Capacity (mAh g^{-1})**	**Current density/Capacity (mAh g^{-1})**
**CFC/S[40]**	33.4%	1.5 M $NaClO_4$ and 0.2 M $NaNO_3$ in TEGDME	0.05 C/491,	0.1 C/141	0.2 C/265	0.5 C/80
**S/CHNBs@PCNFs[12]**	71.2%	1 M $NaClO_4$ in EC/PC with FEC	0.2 C/709	0.5 C/598	1 C/487	------
**S@iMCHS[41]**	46%	1 M $NaClO_4$ in EC/PC+FEC	0.1 C/391	0.2 C/386	0.5 C/352	1 C/305
**S@C[35]**	35%	$NaPF_6$ in TEGDME	0.1 C/900	0.25 C/510	0.5 C/490	1 C/370
**HSMC-Cu-S[23]**	50%	1.0 M $NaClO_4$ EC/ DMC	0.03 C/610	0.06 C/600	0.6 C/400	1.2 C/290
**S@Con-HC[15]**	~47%	1.0 M $NaClO_4$ in PC/EC + 5 wt% FEC)	0.1 C/820	0.2 C/498	0.5 C/383	1 C/313
**S@Co/C/rGO[42]**	37.5%	PFSA-Na membranes	0.1 C/461.2	0.2 C/208.7	0.5 C/164.2	1 C/150.2
**S@Ni-NCFs[27]**	36%	1 M $NaClO_4$ in TEGDME	0.2 C/738.7	0.3 C/565.6	0.5 C/481.1	1.0 C/311.1
**CNF-L@Co/S**	45%	1 M $NaClO_4$ in EC / DEC	0.2 C/929.8	0.5 C/700	1 C/540	1.5 C/442.7

Table S2 Compared all the similar studies in terms of structure and Na-S performance parameters

Cathode structure	Synthetic methods	Sulfur content (%)	Current density	50st cycle capacity (mAh g^{-1})	Refs.
**S@Co nanoparticle decorated hollow carbon nanospheres**	Synthetic method: hard templates and sol-gel process<br>Carbon matrix: hollow carbon nanospheres<br>Cobalt source:$CoCl_2$<br>Carbonization conditions: 200 °C for 2 h in a forming gas with 10 vol% H_2 in nitrogen, leading to the formation of Co-HC.	47%	100 mA g^{-1}	708	[15]
**S@Co/C/rGO cobalt nanoparticles on graphene aerogel**	Synthetic method: sol-gel process and hydro-thermal<br>Matrix：graphene aeroge<br>Cobalt source: $Co(NO_3)_2$ (Aladdin, AR, 99%), 15 mL NH_3•H_2O (Aladdin, AR, 25-28%) and 10 mL H_2O<br>Carbonization conditions: at 400 °C for 2 h in H_2 (H_2% = 5%) atmosphere and another 800 °C for 2 h in Ar atmosphere, the heating rate is 5 °C/min. The Co/C/rGO hybrid was obtained.	37.5%	0.1 C	374	[42]
**Co@NPCNFs/S Cobalt nanoparticles embedded into free-standing carbon nanofibers**	Synthetic method: Electrospinning<br>Matrix：Carbon nanofiber<br>Cobalt source: $Co(NO_3)_2$•$6H_2O$ and 0.72 g PVP were added into 4 mL DMF<br>Carbonization conditions:： at 350 ℃ for 2 h and 800 ℃ for 2 h in Ar atmosphere.	35%	0.1 C	820	[43]
**CNF-L@Co/S "Branch-leaf" Electrode**	Synthetic method：Electrospinning and solution method<br>Matrix：carbon nanofiber<br>Cobalt source: 582 mg $Co(NO_3)_2 \cdot 6H_2O$ and 1300 mg 2-methylimidazole (2-MIM) in 40 mL deionized water.<br>Carbonization conditions：at 350 ˚C for 20 min and continues to raise to 800 ˚C maintains for 2 h under the protection of Ar atmosphere to obtain CNF-L@Co compound.	45%	0.1 C	1050	This work

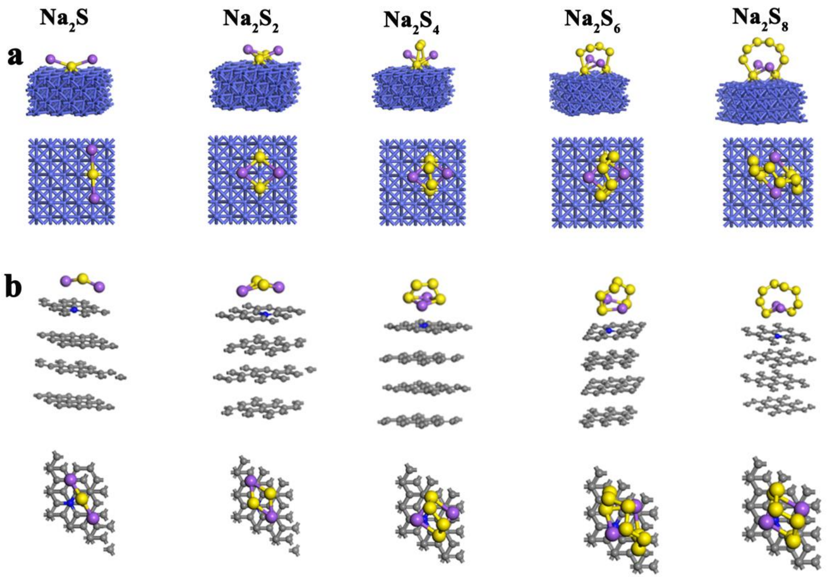

Fig. S14 (**a**) Over Co (111) surface, (**b**) Over the N-doped graphite surface

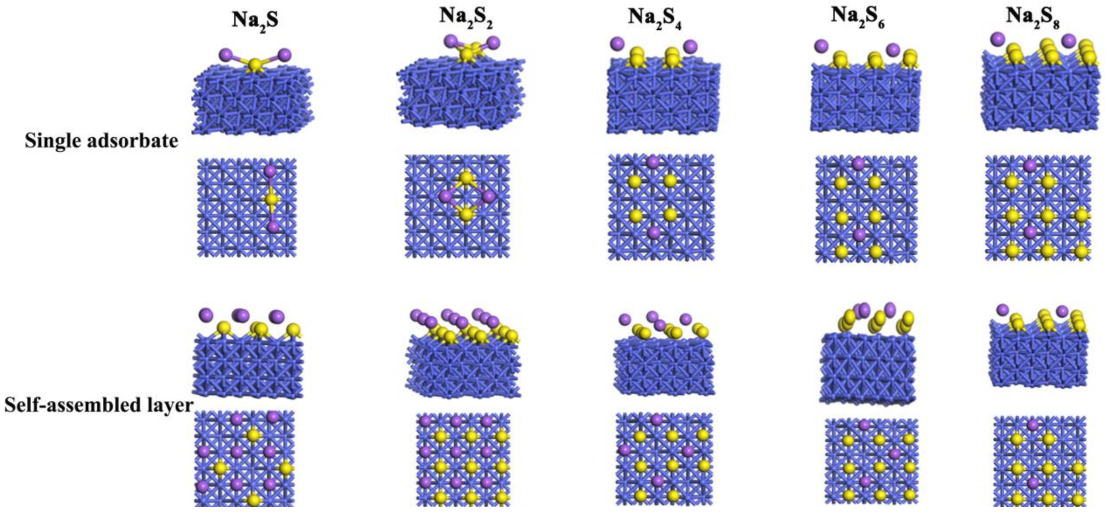

Fig. S15 The most favorable adsorption of Na_2S_x, x = 1-8, at Co (111) surface as single adsorbate and self-assembled layers

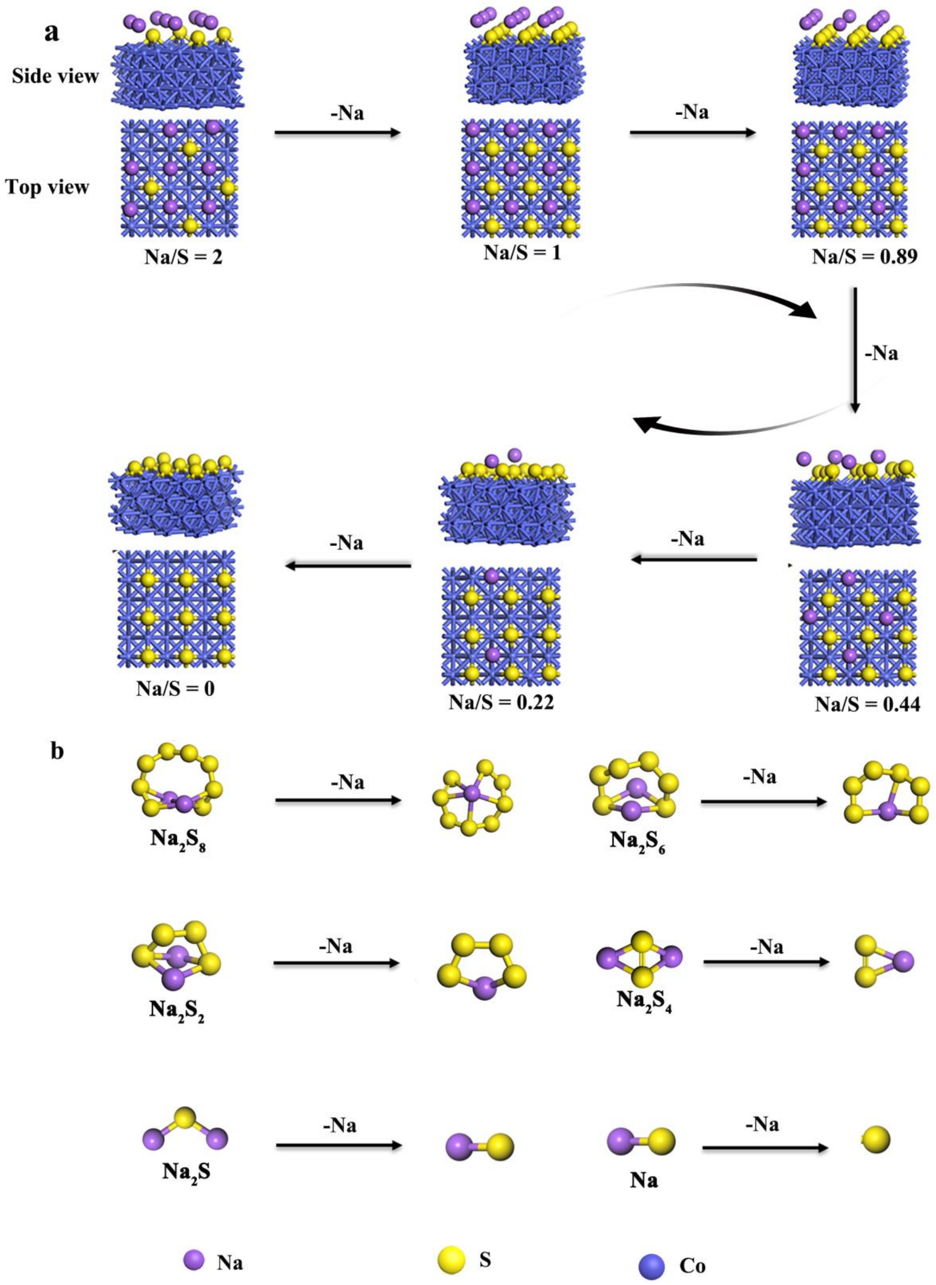

Fig. S16 (**a**) Calculated adsorption energy (eV) for the most favorable adsorption configuration of Na_2S_x at Co (111) surface and N-doped graphite at the DFT level, (**b**) Calculated Na dissociation energy (in eV) at the DFT level for the layered Co-S-Na structures and free Na_2S_x, x = 1-8

Supplementary References

[S1] W. Kohn, L.J. Sham, Self-consistent equations including exchange and correlation effects. Phys. Rev. A **140**, A1133-A1138 (1965). https://doi.org/10.1103/PhysRev.140.A1133

[S2] G. Kresse, J. Furthmüller, Efficient iterative schemes for ab initio total-energy calculations using a plane-wave basis set. Phys. Rev. B **154**, 11169-11186 (1996). https://doi.org/10.1103/PhysRevB.54.11169

[S3] J.P. Perdew, K. Burke, M. Ernzerhof, Generalized Gradient Approximation Made Simple. Phys. Rev. Lett. **77**, 3865-3868 (1996). https://doi.org/10.1103/PhysRevLett.77.3865

[S4] G. Kresse, D. Joubert, From ultrasoft pseudopotentials to the projector augmented-wave method. Phys. Rev. B **59**, 1758-1775 (1999). https://doi.org/10.1103/PhysRevB.59.1758

[S5] H.J. Monkhorst, J.D. Pack, "Special points for Brillouin-zone integrations"—a reply. Phys. Rev. B **13**, 5188-5192 (1976). https://doi.org/10.1103/PhysRevB.16.1748
